# The ligand-bound state of a G protein-coupled receptor stabilizes the interaction of functional cholesterol molecules

**DOI:** 10.1016/j.jlr.2021.100059

**Published:** 2021-02-26

**Authors:** Laura Lemel, Katarzyna Nieścierowicz, M. Dolores García-Fernández, Leonardo Darré, Thierry Durroux, Marta Busnelli, Mylène Pezet, Fabrice Rébeillé, Juliette Jouhet, Bernard Mouillac, Carmen Domene, Bice Chini, Vadim Cherezov, Christophe J. Moreau

**Affiliations:** 1 Univ. Grenoble Alpes, CNRS, CEA, IBS, Grenoble, France; 2 Functional Genomics Laboratory and Biomolecular Simulations Laboratory, Institut Pasteur de Montevideo, Montevideo, Uruguay; 3 Institut de Génomique Fonctionnelle, Université de Montpellier, CNRS, INSERM, Montpellier, France; 4 CNR, Institute of Neuroscience, U28 and NeuroMI Center for Neuroscience, University of Milano-Bicocca, Vedano al Lambro (MB), Italy; 5 Institute for Advanced Biosciences, Inserm U 1209, CNRS UMR 5309, Grenoble Alpes University, Grenoble, France; 6 Laboratoire de Physiologie Cellulaire Végétale, Univ. Grenoble Alpes, CNRS, CEA, INRAE, Grenoble, France; 7 Department of Chemistry, University of Bath, Bath, United Kingdom; 8 Chemistry Research Laboratory, University of Oxford, Oxford, United Kingdom; 9 Bridge Institute, Department of Chemistry, University of Southern California, Los Angeles, CA, USA

**Keywords:** cholesterol, receptors/seven transmembrane domain, receptors/plasma membrane, lipid rafts, cholesterol/physical chemistry, cholesterol binding, oxytocin G protein-coupled receptor, membrane protein-lipid interaction, molecular biology, allosteric regulation, CHS, cholesteryl hemi-succinate, GPCR, G protein-coupled receptor, ICCR, ion channel-coupled receptor, Kir6.2, inward rectifier potassium channel Kir6.2, M2, human M2 muscarinic receptor, MβCD, methyl-β-cyclodextrin, OXTR, oxytocin G protein-coupled receptor, T4L, T4 phage lysozyme, TEVC, two-electrode voltage-clamp, TR-FRET, time-resolved FRET

## Abstract

Cholesterol is a major component of mammalian plasma membranes that not only affects the physical properties of the lipid bilayer but also is the function of many membrane proteins including G protein-coupled receptors. The oxytocin receptor (OXTR) is involved in parturition and lactation of mammals and in their emotional and social behaviors. Cholesterol acts on OXTR as an allosteric modulator inducing a high-affinity state for orthosteric ligands through a molecular mechanism that has yet to be determined. Using the ion channel-coupled receptor technology, we developed a functional assay of cholesterol modulation of G protein-coupled receptors that is independent of intracellular signaling pathways and operational in living cells. Using this assay, we discovered a stable binding of cholesterol molecules to the receptor when it adopts an orthosteric ligand-bound state. This stable interaction preserves the cholesterol-dependent activity of the receptor in cholesterol-depleted membranes. This mechanism was confirmed using time-resolved FRET experiments on WT OXTR expressed in CHO cells. Consequently, a positive cross-regulation sequentially occurs in OXTR between cholesterol and orthosteric ligands.

## Graphical abstract

**Figure:**
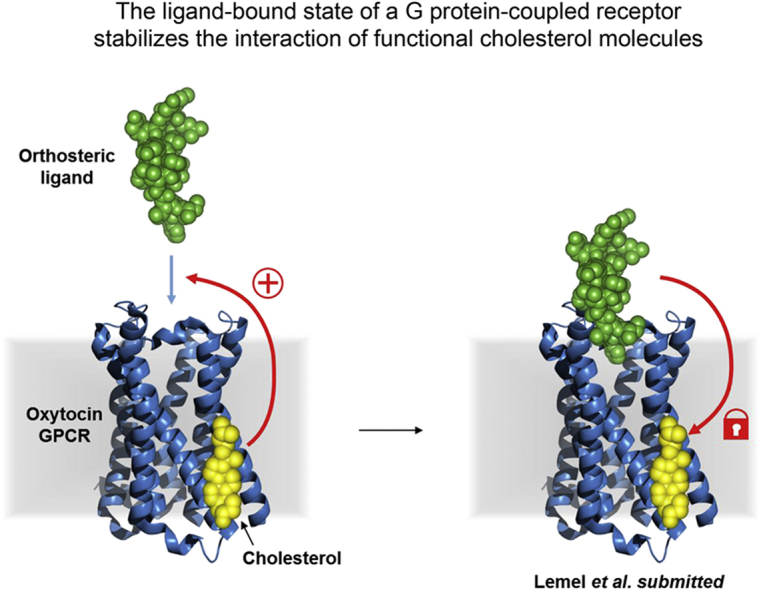

Mammalian G protein-coupled receptors (GPCRs) are transmembrane proteins embedded in cholesterol-containing lipid membranes. Because some GPCRs require the presence of cholesterol for their proper function and the high specificity for this sterol, numerous studies have attempted to decipher the exact molecular mechanisms of this lipid-protein interaction using biochemical, biophysical, structural, and computational studies (1). It is known that cholesterol affects the activity of some GPCRs either by altering the physical properties of the membrane (thickness and/or fluidity) or by interacting directly with the receptor or through a combination of these two effects (2). Distinguishing between these effects on different receptors is not technically trivial and is determined by comparing, in altered and natural cholesterol conditions, diverse parameters such as thermostabilization, protein hydrolysis profile, pH sensitivity (2, 3), and change of membrane fluidity with various steroids (4). In the case of the oxytocin receptor, a comparative analysis of radioligand binding and membrane anisotropy demonstrated an absence of correlation between the cholesterol dependence of oxytocin receptor (OXTR) and the membrane fluidity, which suggests a direct interaction of cholesterol molecules with the receptor (4).

Both the molecular mechanisms of GPCR regulation by cholesterol and the cholesterol binding sites have not been conclusively defined by experimental results. Evidence of direct interaction of cholesterol with GPCRs has been observed in crystal structures of several different receptors (5, 6) such as β_2_AR (PDB code: 2RH1, 3D4S) (7, 8), A_2A_R (4EIY) (9), 5-HT_2B_R (4IB4) (10), μOR (4DKL, 5C1M) (11, 12), P2Y_12_R (4NTJ, 4PXZ) (13, 14), P2Y_1_R (4XNV) (15), mGlu_1_R (4OR2) (16), viral US28 (4XT1) (17), κOR (6B73) (18), ET_B_ (5X93) (19), CB1 (5XRA) (20), CCR9 (5LWE) (21), and SMO (5L7D) (22). The cholesterol sites are distributed in both membrane leaflets (3), and cholesterol molecules are present at interfaces of β_2_AR (2RH1) (7) and mGlu_1_R (4OR2) (16) dimers. Molecular dynamics simulations indicate different exchange kinetics of cholesterol molecules with receptors like β_2_AR depending on their interaction with specific sites (hot spots) (23). A model to differentiate cholesterol molecules with different binding kinetics has been proposed, including annular (bulk) cholesterol molecules that surround GPCRs with fast exchange rates (sub-microsecond time-scale), and nonannular (bound) molecules that tightly bind to the receptor with slow exchange rates (microsecond time-scale) (3, 23, 24, 25). It is still not known whether these bound cholesterol molecules have a functional role, or which binding sites are involved. To identify the binding site(s) of functional cholesterol molecules, the challenge is currently to overcome two obstacles: *i*) the current lack of technology able to isolate functional cholesterol molecules in a membrane containing a majority of nonfunctional cholesterol molecules and *ii*) the lack of stable interactions of functional cholesterol molecules with the receptor because of the frequent exchanges of bulk and bound cholesterol molecules in the microsecond time-scale.

For the OXTR, the focus of this study, six cholesterol molecules have been suggested to play a role in the high-affinity state of the receptor (26). However, their positions could not be precisely determined by receptor mutagenesis or photoaffinity labeling. Moreover, the mechanisms of cholesterol dependence at the molecular level are not yet known (27), and the cholesterol dependence on GPCR function is still not clear for most receptors (28).

In this study, we present a new assay to study the functional cholesterol dependence of GPCRs by sensing in real-time ligand-induced conformational changes of the receptors in a natural membrane environment in living cells. This assay is based on the ion channel-coupled receptor (ICCR) technology. ICCRs are created by genetic fusion of GPCRs to a potassium channel (Kir6.2) (29). The ion channel acts as a real-time reporter of conformational changes of the GPCR by generating an electrical signal that can be easily detected by conventional electrophysiological techniques (30) or by nanoelectronic and microelectronic systems (31). ICCRs can detect GPCR ligand binding of orthosteric agonists and antagonists in a concentration-dependent manner and independently of intracellular signaling pathways (29, 32).

The OXTR was chosen as a cholesterol-dependent GPCR model for this study to assess the ability of the ICCR technology to detect cholesterol-dependence of GPCRs. The receptor is involved in various physiological functions related to pregnancy such as uterine contractions (33) and lactation (34). It is also involved in social behavior and is consequently a potential target for treating neuropsychiatric disorders (35) including autism, schizophrenia and anxiety (36).

Cholesterol acts on OXTR as an allosteric regulator required for the high affinity state of the receptor (Kd ∼1 nM for oxytocin), which exists in equilibrium with a low affinity state (Kd∼100 nM). It is suggested that high and low affinity states are conformationally different, as cholesterol binding induces a more compact and less dynamic state with an increase of the thermal stability of the receptor (37). The molecular mechanisms of this dynamic process induced by bound cholesterol molecules is still unknown.

The oxytocin ICCR has been designed in a previous study (32), where it was heterologously expressed in Xenopus oocytes and functionally characterized by the two-electrode voltage-clamp (TEVC) technique. This technique records real-time whole cell currents generated by ICCRs present in the plasma membrane. External ligands can be easily applied in various concentrations. In Xenopus oocytes, cholesterol is endogenously present in the plasma membrane of these giant cells (∼1 mm in diameter), and its concentration is estimated to be 20.7 mol % (38), which is of the same order of magnitude as the concentrations found in most human cells (28 mol %) (39). OXTR is coupled to Gi/o and Gq proteins (40), both proteins being endogenous in Xenopus oocytes. The activation of the Gq protein signaling pathway leads to (i) activation of problematic effectors for TEVC recordings, namely endogenous calcium-activated chloride channels generating very large interference currents and (ii) the closure of the fused Kir6.2 channel in ICCR because of the decrease of phosphatidylinositol-4,5-bisphosphate concentration in the plasma membrane. To prevent these effects, inhibitors of Gq proteins can be applied (41) (42). In this work, however, we took advantage of a previously designed ICCR in which OXTR has been uncoupled from G proteins by replacing the third intracellular loop with the T4 phage lysozyme (T4L) domain (32). We showed that the OXTR(T4L) ICCR reported the receptor activity independently of any intracellular signaling pathways.

Using this assay, we discovered an unreported mechanism of stabilization of the interaction of functional cholesterol molecules with OXTR when the receptor adopts a ligand-bound state. These results highlight a simple method to discriminate and isolate functional bound cholesterol molecules in a cell membrane that is naturally abundant in cholesterol. This original functional selection of cholesterol molecules offers new possibilities (i) for the challenging identification of their binding site(s) by structural, biochemical and computational studies, (ii) for deciphering the molecular mechanisms of the allosteric cross-regulation occurring between the cholesterol and the oxytocin binding sites, and (iii) for evaluating the role of the stable oxytocin-bound and cholesterol-bound state of the receptor in physiological processes where this state takes place (recycling, intracellular receptor signaling).

## Materials and methods

### Molecular biology

All genes were subcloned in pGEMHE-derived vectors optimized for protein expression in Xenopus oocytes (32). After cDNA linearization in the 3′ end of the polyA tail, mRNA was synthesized using the T7 mMessage mMachine Kit and purified by the standard phenol:chloroform protocol, analyzed by agarose-gel electrophoresis and quantified by spectrophotometry (43). Kir6.2 is truncated of its last 36 residues (Kir6.2ΔC36) to remove a known endoplasmic reticulum retention signal and to allow the surface expression of the channel alone (44) or the T4L-modified ICCRs (32). The T4L domain was inserted between QNL^231^ [T4L] ^264^KLI in OXTR and between SRI^217^ [T4L] ^377^PPP in the human M2 muscarinic acetylcholine receptor (M2). In the OXTR-ICCR, the last 42 residues of OXTR were truncated to create a functional coupling between the receptor and the fused ion channel (32).

### Reagents

Oxytocin was acquired from GenScript. Atosiban and SR49059 were purchased from Bachem (UK). Methyl-β-cyclodextrin (MβCD) (C4555), filipin (F9765), and digitonin (D141) were purchased from Sigma-Aldrich. RS544-red was prepared as previously reported (45), and the fluorophore was modified to d2 by the Cisbio company.

### Electrophysiological recordings

Xenopus oocytes were prepared as previously reported (29). Animal handling and experiments fully conformed to European regulations and were approved by the French Ministry of Higher Education and Research (APAFIS#4420-2016030813053199 v4 to CM). Authorization of the animal facility has been delivered by the Prefect of Isere (Authorization # D 38 185 10 001). Amounts of mRNA injected per oocyte and coding for the following proteins were: ICCR 4 ng, OXTR 2 ng, Kir6.2ΔC36 2 ng. TEVC recordings were initially performed manually and later automatically with the HiClamp robot (Multi Channel Systems). During recordings, oocytes were incubated in high potassium buffer: 91 mM KCl, 1.8 mM CaCl_2_, 1 mM MgCl_2_, 5 mM HEPES, 0.3 mM niflumic acid, pH 7.4. Ligands and BaCl_2_ are diluted in this high potassium buffer. The membrane voltage was clamped at −50 mV.

### Cholesterol manipulation

Cholesterol depletion was performed by incubation of Xenopus oocytes in 96-well plates in 200 μl of 20 mM MβCD in modified Barth's solution (30) for at least 3 h at 19°C. Cholesterol repletion was performed by re-incubating cholesterol-depleted and nonrecorded oocytes in 40 mM cholesterol, lanosterol, or cholesteryl hemi-succinate (CHS) solubilized with 5 mM MβCD for 1 h at 19°C. Experiments of cholesterol depletion in presence of ligands were performed by adding 5 μM of oxytocin to the modified Barth's solution containing 20 mM MβCD and then incubated for at least 3 h at 19°C. To wash bound ligands before testing ICCR function, oocytes were placed for 2 min in a constant flow of high potassium buffer. A second incubation of 20 mM MβCD was performed on oocytes preincubated for 3 h in 20 mM MβCD + 5 μM oxytocin. Before the second incubation, the oocytes were pooled in 15 ml-tube and washed 3 times 5 min in the modified Barth's solution to remove the ligand. Oocytes were reloaded individually in wells of a 96-well plate filled with 200 μl of 20 mM MβCD in modified Barth's solution and incubated for 1 h at 19°C.

### Filipin-fluorescence microscopy

Filipin 0.05% (w/v) was added to wells containing oocytes, 1 h before the end of the 3 h-incubation with MβCD or buffer, with and without 1 μM oxytocin. Confocal microscopy was performed on a Zeiss inverted LSM710 microscope equipped with a 40× N.A.1.20 C-Apochromat water immersion lens (Carl Zeiss MicroImaging GmbH, Germany). Images were acquired at the equatorial plane focused in brightfield mode. The filipin signal was collected using a two-photon laser at 700 nm and low power (2%) to avoid photobleaching and emission set to 400–485 nm. Gray intensities of pixels were analyzed with ImageJ using the integrated pixel density parameter and background subtraction with a threshold of 200 on a gray scale from 0 to 1,403.

### Digitonin assay

Cholesterol-depletion was performed as described in the section “Cholesterol Modification”. Xenopus oocytes, preincubated for 3 h in 20 mM of MβCD or in Buffer (modified Barth's solution), were incubated in 10 μM digitonin for 2 min in low-potassium buffer (ND96 buffer): 96 mM NaCl, 2 mM KCl, 1.8 mM CaCl_2_, 1 mM MgCl_2_, 5 mM HEPES, 0.3 mM niflumic acid, pH 7.4. Currents were recorded in real-time with the TEVC HiClamp automate. Traces are the average ± SEM of 7 or 9 recorded oocytes incubated with buffer or MβCD, respectively.

### Time-resolved FRET experiments on WT OXTR in CHO cells

CHO cells were transfected according to the manufacturer's recommendation (jetPEI DNA transfection, Polyplus-transfection, Illkirch, France). Briefly, cells were seeded on day 1 in 6-well plates at a concentration of 300,000 cells/well. On day 2, 6 μl/well of JetPEI diluted in 100 μl NaCl (150 mM) were added to a mix of DNA coding for SNAP-OXTR (180 ng/well) and uncoding DNA (2820 ng /well) diluted in 100 μl NaCl (150 mM). The mixture was incubated for at least 30 min at room temperature and was then added onto the cells. On day 3, cells in the 6-well plates were then harvested after addition of trypsin, counted and seeded at a concentration of 30,000 cells/well in a white 96-well plates. Experiments were carried out on day 4. Cells were labeled with SNAP-Lumi4-Tb (100 nM) (Cisbio Bioassays, Codolet, France) at 37°C for 1 hour, rinsed four times with Tag-lite buffer (Cisbio Bioassays) and incubated in the presence of the various compounds. The time-resolved FRET (TR-FRET) signal was measured on a Pherastar (BMG Labtech). Cells were illuminated at 337 nm, and luminescent signals were measured at 620 nm and 665 nm every minute. The ratio (665/620) was then plotted as a function of time. Experiments were performed three times, independently.

## Results

### The ICCR technology detects the cholesterol dependence of OXTR

**Fig. 1:**
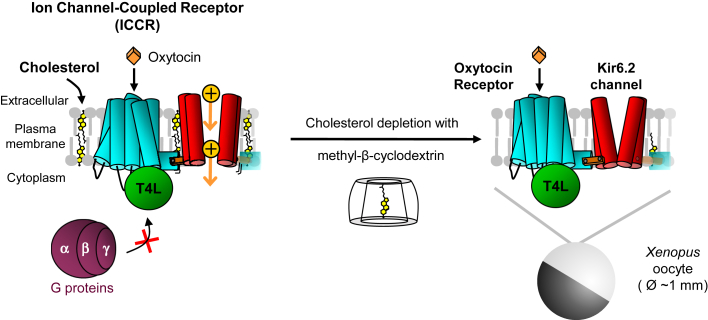
Functional characterization of cholesterol-dependence of G protein-coupled receptor with the ion channel-coupled receptor technology. The ion channel-coupled receptor (ICCR) is created by linking the C-terminus of the oxytocin receptor (OXTR) to the N-terminus of the Kir6.2 channel. Kir6.2 homotetramerizes forming a potassium selective pore. Binding of oxytocin induces conformational changes inducing an increase of the current amplitude generated by the ion channel. The ICCR is heterologously expressed in Xenopus oocytes, and endogenous cholesterol is depleted by incubation with methyl-β cyclodextrin. The proteins are modified as follow: the C-terminus of OXTR is truncated of the last 42 residues, and the third intracellular loop is replaced by the T4 phage lysozyme (T4L) domain that prevents G proteins binding; the N-terminus of Kir6.2 is truncated of its first 25 residues. Currents generated by the ion channel are recorded with the two-electrode voltage-clamp technique in high external K^+^ concentration (91 mM) and a membrane voltage clamped to −50 mV inducing an inward flow of K^+^ ions (yellow circles).

The oxytocin ICCR was previously reported to activate the fused Kir6.2 ion channel in presence of oxytocin (32). To assess whether the ICCR technology is able to detect the cholesterol dependence of the OXTR, the endogenous cholesterol present in the Xenopus oocytes plasma membrane (38) was depleted by incubation with MβCD (Fig. 1).

**Fig. 2:**
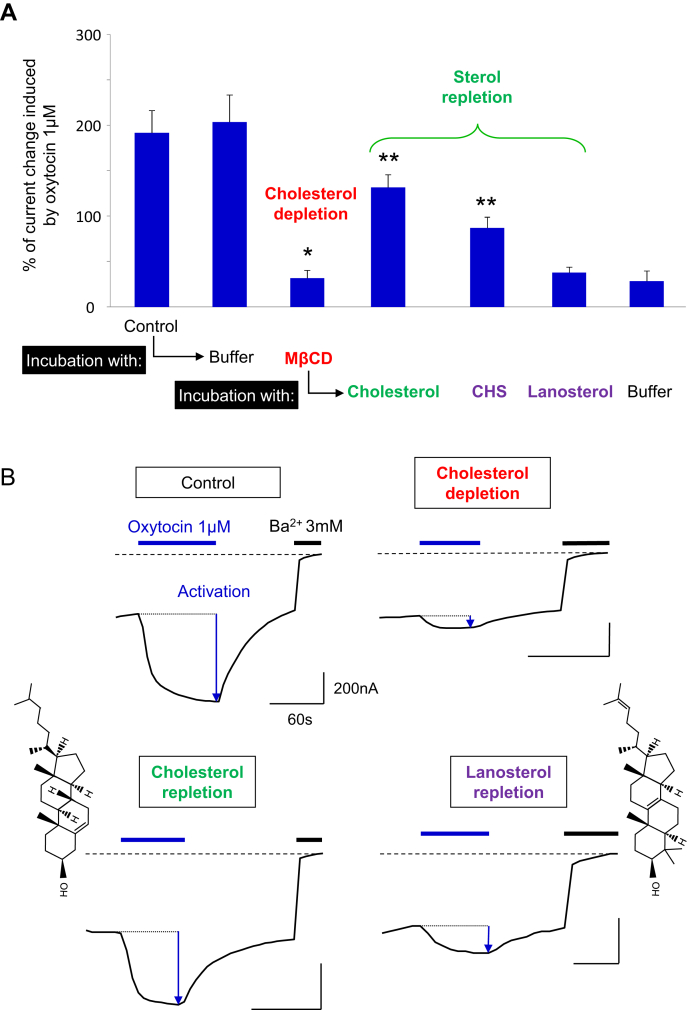
The ICCR technology reports the cholesterol-dependence of the OXTR. A: Histogram showing the mean ± SEM of the percentage of current change induced by 1 μM of oxytocin and measured by TEVC recordings on ICCR-expressing Xenopus oocytes in various conditions indicated in abscissa. Initial Control is performed in high external K^+^ buffer. Oocytes are incubated for 3 h either in modified Barth's buffer (Buffer) or in 20 mM methyl-β cyclodextrin (MβCD) for cholesterol depletion. Nonrecorded oocytes incubated with MβCD are subsequently incubated for 1 h with 40 mM cholesterol, cholesterol hemisuccinate (CHS), or lanosterol, solubilized in 5 mM MβCD, for sterol repletion or with Buffer as negative control. The number of recordings (n) is between 8 and 141. *P* values are measured with the Student *t* test. ∗*P* < 0.0001 (ref= Control); ∗∗*P* < 0.0001 (ref = MβCD). B: Representative TEVC recordings showing the current induced by 1 μM oxytocin after incubation of oocytes in the indicated conditions. By convention, the current is negative, and an increase of the current amplitude results in a downward deflection. Barium (Ba^2+^) is used as a potassium channel blocker and defines the baseline in dashed line. Chemical structures of cholesterol and lanosterol are shown on left and right respectively. ICCR, ion channel-coupled receptor; OXTR, oxytocin receptor; TVEC, two-electrode voltage-clamp.

MβCD is a water-soluble cyclic oligosaccharide composed of seven molecules of methylated glucose forming a central hydrophobic cavity with a high specificity for cholesterol. Incubation of living cells with methyl-β-cyclodextrin induces depletion of cholesterol from the plasma membrane by solubilizing the cholesterol molecules (46). Figure 2 illustrates the activation of OXTR by 1 μM oxytocin in terms of relative current amplitudes generated by the fused ion channel. The results demonstrate that cholesterol depletion (MβCD in Fig. 2A) leads to a large reduction of ICCR activation induced by 1 μM oxytocin.

In contrast, when oocytes are not depleted in cholesterol but incubated with cyclodextrin-free buffer (Buffer in Fig. 2A), the amplitude of activation remains significantly similar to the control before incubation. MβCD being not strictly specific to cholesterol, a standard control consists in re-incubating depleted membranes with cholesterol only. This was performed with cholesterol solubilized in saturated MβCD (47) incubated with oocytes previously depleted in cholesterol. The ICCR activation is significantly, but not totally restored (∼70% of the control before incubation) by cholesterol repletion (Incubation with: Cholesterol in Fig. 2A). This is in agreement with binding experiments on HEK293 cells showing restoration of 70%–100% of high-affinity oxytocin binding after cholesterol repletion (4). Additional controls were set up without cholesterol (Buffer in Fig. 2A) and with two cholesterol analogs: (i) the CHS, widely used as cholesterol-surrogate in biochemical and structural studies because of its higher solubility in aqueous solutions(48), (23) and (ii) lanosterol, a natural precursor of cholesterol synthesis. Re-incubation with buffer did not restore the ICCR activation indicating that endogenous cholesterol replenishment is absent or weak under our experimental conditions. Similarly, membrane repletion with lanosterol did not restore the ICCR activation which was expected because lanosterol restored only 7% of high-affinity oxytocin binding in HEK293 cells (4). In contrast, CHS was able to partially restore the activation of the ICCR(∼45% of the control level before incubation), confirming its role as a functional cholesterol substitute for OXTR. While the functional effect of CHS on the restoration of ICCR activation is clear and indicate a partition of CHS in the plasma membrane, its quantitative interpretation on the amplitude of activation must be taken with care in absence of CHS concentration measurements in Xenopus oocytes.

These results demonstrate that the loss of activation after MβCD incubation was caused specifically by cholesterol depletion. Furthermore, they confirm that the ICCR technology detects cholesterol dependence of OXTR.

### The Kir6.2 channel is not involved in ICCR cholesterol sensitivity

**Fig. 3:**
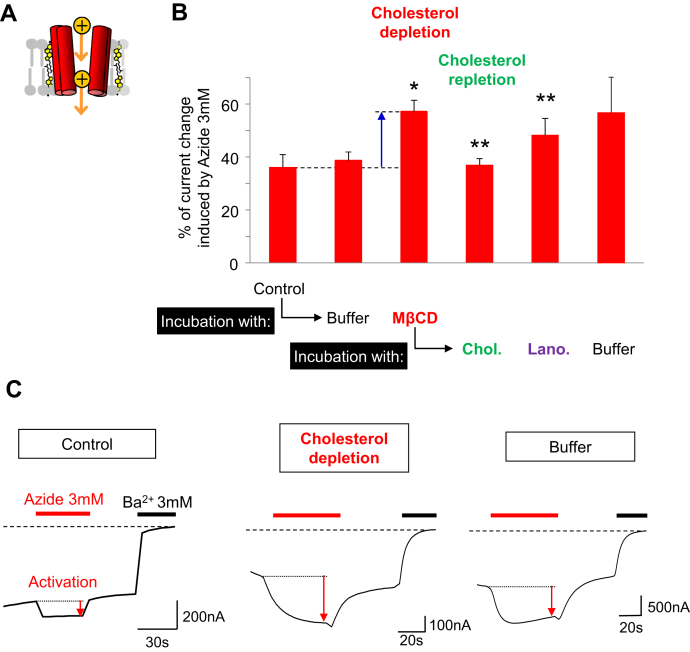
Effect of cholesterol on the ion channel Kir6.2. A: Diagram of the Kir6.2 channel alone for indicating that experiments are performed only on the ion channel. Front and back subunits are hidden, and surrounding cholesterol molecules are shown. In experimental conditions (91 mM external K^+^ and Vm = −50 mV), the K^+^ flow is inward. B: Histogram showing the mean ± SEM of the percentage of current change induced by 3 mM of azide and measured by TEVC recordings on ICCR-expressing Xenopus oocytes in the various conditions indicated in abscissa. Cholesterol (Chol.) and lanosterol (Lano.) are applied as described in the legend of Fig. 2. The number of recordings (n) is between 6 and 33. *P* values are measured with the Student *t* test. ∗*P* < 0.0001 (ref= Control); ∗∗*P* < 0.0001 (ref= MβCD). C: Representative TEVC recordings showing the current induced by 3 mM of azide after incubation of oocytes in the indicated conditions. ICCR, ion channel-coupled receptor; MβCD, methyl-β cyclodextrin; TVEC, two-electrode voltage-clamp.

While MβCD has the highest affinity for cholesterol among the cyclodextrins, it can also extract other lipids that could decrease the ion channel activation. Moreover, cholesterol is also known to affect the activity of ion channels such as Kir6.2 (49). To prove that the loss of ICCR activation under cholesterol-depleted experiment is not a result of loss of ion channel function, the same cholesterol-depletion experiment was performed on the ion channel only. Under physiological conditions, Kir6.2 must form an octameric complex with the sulfonylurea receptor to traffic to the plasma membrane. However, deletion of an endoplasmic reticulum retention signal in Kir6.2 C-terminus (Kir6.2ΔC36) enables the surface expression of the homotetrameric Kir6.2 channel alone (44). Figure 3 shows that, in contrast to the experiments with ICCR, cholesterol depletion increases the activation of Kir6.2ΔC36 by extracellular sodium azide (50). Cholesterol repletion and partially lanosterol repletion restored the initial amplitude of activation by azide while the control with buffer did not. These results are in agreement with previous studies showing an inhibition of Kir6.2 by cholesterol (49). Consequently, the loss of ICCR activation cannot be attributed to a direct effect on the ion channel.

### The cholesterol dependence of the ICCR is specific to the oxytocin receptor

**Fig. 4:**
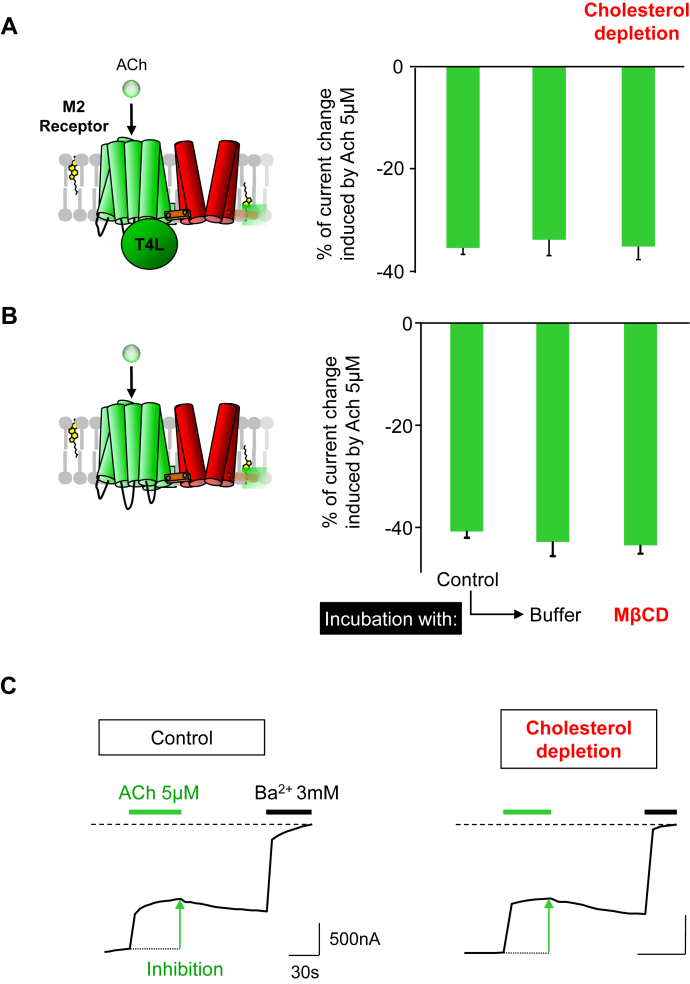
Cholesterol has no effect on the M2 muscarinic ICCR. A: Diagram of the M2 muscarinic ICCR with the T4 phage lysozyme (T4L) domain in place of the third intracellular loop (32). This construct (M2=K-9-25) (43) is inhibited upon binding of the agonist acetylcholine (ACh). Histogram showing the mean ± SEM of the percentage of current change (inhibition) induced by 5 μM of ACh and measured by TEVC recordings on ICCR-expressing Xenopus oocytes in various conditions indicated in panel B. The number of recordings (n) is between 7 and 25. B: Same representation with the M2 muscarinic ICCR without T4L domain. The number of recordings (n) is between 14 and 24. C: Representative TEVC recordings showing the current induced by 5 μM ACh after incubation in the indicated conditions of oocytes expressing M2=K-9-25 without T4L domain. ICCR, ion channel-coupled receptor; T4L, T4 phage lysozyme.

To verify that the cholesterol effect was specific to the OXTR, the receptor was replaced by the human muscarinic M2 receptor. The M2 receptor has not been reported as cholesterol-dependent (28) for its activity, and no cholesterol molecules have been observed in its different crystal structures (3UON (51), 4MQS (52) and 6OIK (53)). Two M2 ICCRs were used for the experiment: *i*) the T4L version (32), as in the OXTR ICCR and *ii*) the M2 receptor with an intact third intracellular loop, which was possible to use because the M2 receptor is not coupled to the problematic Gq proteins. Both ICCRs were engineered to be inhibited by the agonist acetylcholine (43). The functional characterization of M2(T4L)-ICCR showed that the amplitude of the inhibition is unchanged after incubation with MβCD or with buffer (Fig. 4A). Consequently, the cholesterol depletion did not affect the M2 receptor function. To verify that the exogenous T4L domain did not alter potential cholesterol sensitivity of the M2 receptor, the same experiments were performed on the M2-ICCR with an intact third intracellular loop. The same results were observed (Fig. 4B), indicating that the T4L domain has no influence on the cholesterol-insensitivity of the M2 receptor.

These results confirm that: *1*) the cholesterol dependence observed for the OXTR-ICCR is specific to the OXTR and *2*) the human M2 muscarinic receptor is not cholesterol dependent for its activity, which is in agreement with previous results showing a similar activity of the purified M2 receptor in presence or absence of cholesterol (54).

### The ligand-bound state preserves the oxytocin receptor activity in cholesterol-depleted condition

The ICCR technology used in Xenopus oocytes allows long periods of ligand incubations without internalization (55). Moreover, the replacement of the third intracellular loop by the T4L domain prevents the activation of intracellular signaling pathways. In contrast, in HEK293T mammalian cells, OXTR activation induces fast and almost complete internalization of the receptors (56).

**Fig. 5:**
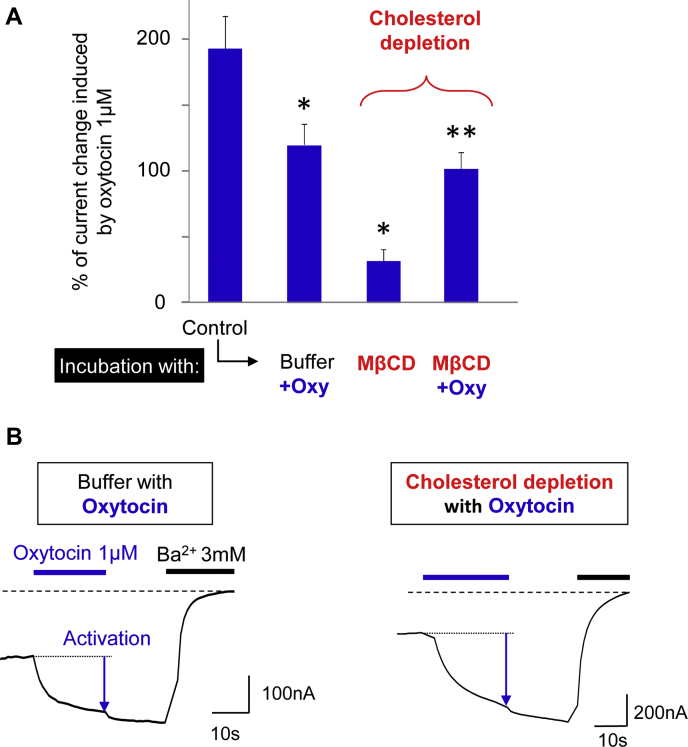
Oxytocin preserves the activity of the OXTR in depleted-cholesterol conditions. A: Histogram showing the mean ± SEM of the percentage of current change induced by 1 μM of oxytocin on the ICCR in the conditions indicated in abscissa. Oxytocin (Oxy) 5 μM is incubated with 20 mM MβCD or buffer for at least 3 h before TEVC recordings of oxytocin-induced activation of the ICCR. The number of recordings (n) is between 6 and 18. *P* values are measured with the Student *t* test. ∗*P* < 0.0005 (ref= Control); ∗∗*P* < 0.0001 (ref= MβCD). B: Representative TEVC recordings showing the current induced by 1 μM oxytocin after incubation of oocytes in the indicated conditions. ICCR, ion channel-coupled receptor; MβCD, methyl-β cyclodextrin; OXTR, oxytocin receptor; TVEC, two-electrode voltage-clamp.

Taking advantage of the Xenopus oocyte characteristics, the OXTR-ICCR was incubated simultaneously with the agonist oxytocin and with MβCD to keep the receptor in agonist-bound state during cholesterol depletion. After washing the MβCD- and oxytocin-containing solution, the activity of the ICCR was measured as in previous experiments by TEVC recordings in presence of oxytocin (Fig. 5). Surprisingly, we observed that the ICCR did not lose its activity, but instead kept an amplitude of activation of 100% of the basal current that is similar to the control without MβCD (Buffer+Oxy, 120%). This result indicates that the agonist-bound state of the receptor preserved its cholesterol-dependent function despite the cholesterol depletion by MβCD.

The activity of the OXTR-ICCR being highly dependent on cholesterol suggests that functional cholesterol molecules are preserved during cholesterol depletion when OXTR adopts an oxytocin-bound state.

Two mechanisms could explain why ligand-bound receptors would stably bind specific cholesterol molecules and make them inaccessible to MβCD: *1*) co-incubation of oxytocin with MβCD hinders cholesterol depletion or *2*) the ligand-bound state of the receptor induces conformational changes that drastically slow down the dissociation kinetics of functional cholesterol molecules resulting in their sequestration and the preservation of the ICCR activity.

**Fig. 6:**
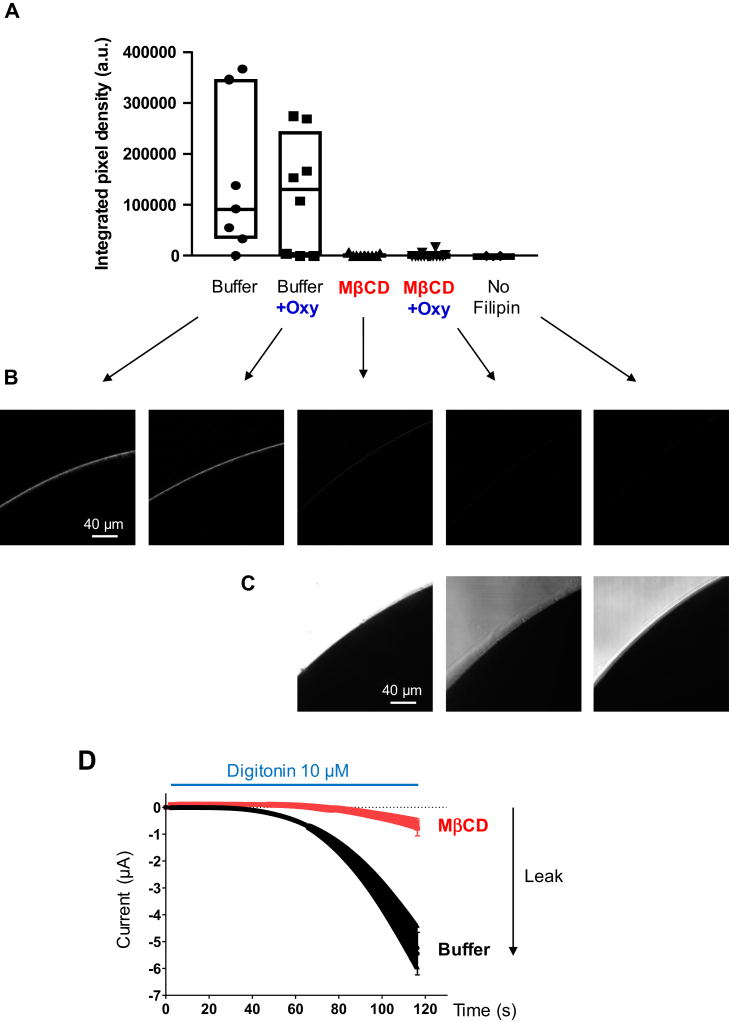
Plasma membrane cholesterol-depletion by methyl-β cyclodextrin in the presence and absence of ligand. A: Dotplot relative to the fluorescence of the cholesterol-probe filipin on Xenopus oocytes expressing the OXTR ICCR and after incubation in the conditions indicated in abscissa. Values are calculated as integrated pixel densities measured by ImageJ on a gray scale from 0 to 1,403 and after background subtraction with a threshold of 200. Boxes are delineated by lower and upper quartiles (Q1 and Q3 respectively) separated by the median (Q2). B: Fluorescence images of filipin-stained Xenopus oocytes at the equator in the conditions indicated by the arrows. Gray level images were acquired at 512 × 512 pixels (0.415 μm/pixel), 12 bits, line averaging 8. Excitation wavelength is 700 nm (2-photon laser) and the emission wavelengths are 405–485 nm. C: Control in brightfield images of oocytes without fluorescence. D: Average of currents recorded by TEVC during application of 10 μM of digitonin to oocytes preincubated with 20 mM MβCD or buffer for 3 h. Digitonin destabilizes the membrane in presence of cholesterol creating an ion leak that is recorded in real-time by TEVC method. The number of recordings is 7 or 9 for the oocytes preincubated with buffer or MβCD, respectively. Error bars are SEM. ICCR, ion channel-coupled receptor; MβCD, methyl-β cyclodextrin; OXTR, oxytocin receptor; TVEC, two-electrode voltage-clamp.

To assess the possibility of a lack of cholesterol extraction by MβCD in our experimental conditions, three controls were carried out: *1*) Confocal fluorescence microscopy was performed on Xenopus oocytes incubated in MβCD with and without oxytocin and stained with the filipin probe. Filipin specifically interacts with cholesterol molecules (57) in the lipid bilayer (58) resulting in an increase of fluorescence intensity at 385–470 nm. Fluorescence images were taken at the equatorial plane of the Xenopus oocytes (Fig. 6), and they demonstrated that MβCD efficiently depleted cholesterol molecules even in the presence of the ligand; *2*) A second approach based on digitonin was used for confirming cholesterol-depletion by MβCD. Digitonin is a saponin from *Digitalis purpurea* which forms digitonin-cholesterol complexes leading to rapid membrane leakage or rupture (59). During TEVC recordings, digitonin at 10 μM was applied to oocytes preincubated with MβCD or with Buffer. The results (Fig. 6D) demonstrated that the leak induced by digitonin was significantly reduced by MβCD preincubation compared with the control with buffer (−0.782 ± 0.283 μA vs. −5.451 ± 0.794 μA at 116.4 s, respectively), indicating that cholesterol was depleted from the plasma membrane; *3*) a third approach attempted to quantify cholesterol after MβCD incubation. However, the isolation of Xenopus oocyte plasma membrane is made complicated by the presence of large intracellular lipid stocks, and only four articles in our knowledge described the procedures (38), (60), (61), (62). All of them were tested, and the protocol adapted from (61) was selected as it provided the best results for plasma membrane isolation in our conditions (supplemental Fig. S1A). Western-blot targeting the Xenopus plasma membrane Cl_Ca_ channel was used to normalize the quantity of isolated plasma membrane between the sample incubated with MβCD and the control sample incubated with Buffer overnight. The results confirmed a similar quantity of this protein, and therefore of plasma membrane, between both samples (supplemental Fig. S1B). Cholesterol quantification with a commercial enzymatic assay showed a decrease of 39.8% of quantity of cholesterol in the sample incubated with MβCD compared with the control (supplemental Fig. S1C). This value must be considered as an estimation because potential contamination with internal lipid stocks could occur. All these approaches confirmed previous reports showing the depletion of cholesterol molecules by MβCD independently of their location in cholesterol-rich or poor domains (63, 64).

Consequently, these results are in opposition to the first hypothesis of impaired cholesterol-depletion.

### Cholesterol molecules stably bind to ligand-bound OXTR

**Fig. 7:**
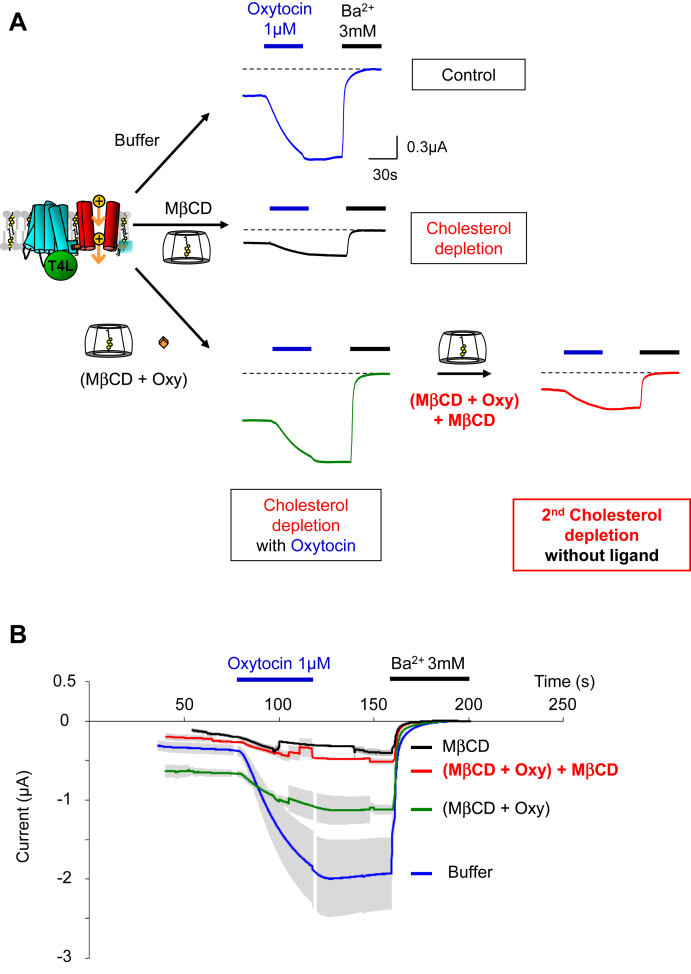
Bound cholesterol molecules are preserved from depletion by ligand-bound receptor. A: Representative TEVC recordings showing the current induced by 1 μM oxytocin after incubation of oocytes in the indicated conditions. The concentration of MβCD is 20 mM, and the concentration of oxytocin (Oxy) during incubations is 5 μM. Incubation time is at least 3 h except for the second cholesterol depletion which is at least 1 h. The second cholesterol depletion is performed from nonrecorded oocytes preincubated with MβCD + Oxy. These oocytes are pooled in 15 ml tube and wash three times 5 min with modified Barth's solution to remove the ligand (Oxy). The washed oocytes are incubated for at least 1 h with MβCD before recordings. B: Average curves ± SEM of traces shown in panel a. The number of recordings (n) is between 3 and 9. MβCD, methyl-β cyclodextrin; TVEC, two-electrode voltage-clamp.

In the second hypothesis, the ligand-bound state of OXTR stabilizes the binding of functional cholesterol molecules. Dissociation of the ligand by washing releases these cholesterol molecules and makes them accessible to MβCD for their extraction from the membrane. To test this hypothesis, oocytes preincubated with MβCD+oxytocin were washed three times for 5 min in 15 ml of modified Barth's solution to dissociate oxytocin ligand from the receptors. The oocytes were re-incubated for at least 1 h in MβCD (without ligand) to extract the potentially released cholesterol molecules. The results (Fig. 7) demonstrate that this second incubation with MβCD (red traces) restored the cholesterol-depleted phenotype of the ICCR as observed in the control (black traces). Consequently, ligand washing allowed the depletion of functional cholesterol molecules that were inaccessible in ligand-bound OXTRs. These results confirm the second hypothesis that the ligand-bound state of OXTR stabilizes functional cholesterol molecules and preserves them from MβCD extraction. This maintains the cholesterol-dependent activity of the ICCR even in cholesterol-depleted membranes. Dissociation of ligands generates ligand-free receptors, which release these specific, functional cholesterol molecules, making them accessible again for extraction by MβCD.

In the case of OXTR, and only when it adopts a ligand-bound state, the interaction of functional cholesterol molecules is highly stable, both during the 3 h of co-incubation with MβCD and ligand and during the initial steps of recordings in buffer.

### In wild-type OXTR, the ligand-bound conformation also preserves the high-affinity state of the receptor in cholesterol-depleted condition

**Fig. 8:**
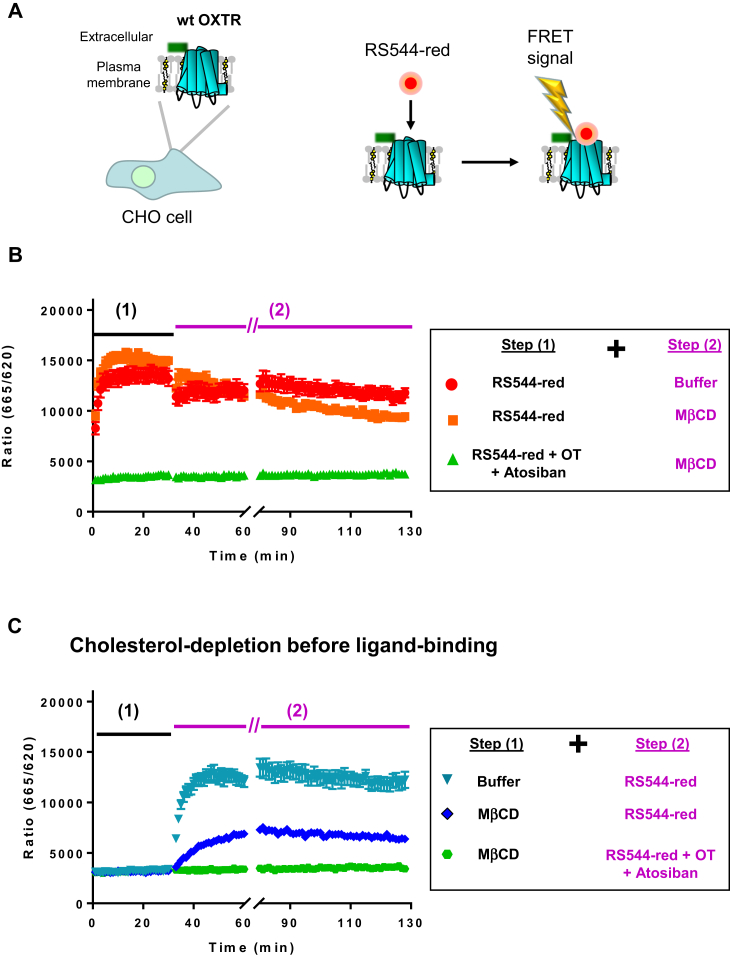
The activity of wild-type OXTR is also preserved in the ligand-bound form during cholesterol depletion in mammalian cells. A: Diagram showing wild-type OXTR heterologously expressed in CHO cells. The receptor has a SNAP tag in N-terminus that binds the cryptate of terbium, Lumi4-Tb. The fluorescent antagonist RS544-red binds to the receptor and generates a FRET signal that is recorded in real-time (time-resolved FRET, TR-FRET). B: TR-FRET signal (ratio of light emission intensity at 665 nm over the intensity at 620 nm) as a function of time in the indicated conditions. Each point is an average of 12 measurements. Cells are placed in wells in 96-well plates and incubated for 30 min with solutions indicated in step 1 in the legend. The solutions of Step 2 are added to the wells for additional incubation of 30 min + 60 min. To avoid dynamic and unspecific FRET, RS544-red was used at 20 nM. The concentration of MβCD was increased to 40 mM because of the shorter incubation time (30 min). Co-incubation with 5 μM of unlabeled Atosiban and Oxytocin were used to determine the level of nonspecific FRET. C: Same conditions of experiments with the solutions of Step 1 and 2 indicated in the legend. MβCD, methyl-β cyclodextrin; OXTR, oxytocin receptor.

To explore the protective effects of ligands on WT OXTR in mammalian cells, TR-FRET experiments were performed on CHO cells transiently expressing WT OXTR. A Snap-Tb fluorescent tag was present at the N-terminus of the receptor, and FRET signal was detected between the tag and bound fluorescently labeled ligands (65) (66) (Fig. 8). We chose an antagonist, RS544-red, to avoid receptor internalization during ligand incubation. Diagrams illustrating the experimental conditions used for TR-FRET recordings are shown in supplemental Fig. S2.

In conditions of cholesterol depletion in presence of ligand (Fig. 8B, orange squares), the FRET signal retains the same amplitude as the controls without cholesterol depletion (Fig. 8B, red dots or Fig. 8C, teal triangle). This result indicates that the ligand-bound conformation of the receptor preserves its cholesterol-dependent high-affinity state even in cholesterol-depleted conditions. When cholesterol depletion was performed in absence of a ligand (Fig. 8C, dark blue diamonds), the FRET signal had a slower kinetics and a lower amplitude indicating a lower affinity state of the receptor, as previously observed (67). Consequently, these results confirmed the ligand-induced preservation of the OXTR high affinity state on the wild-type receptor.

## Discussion

The ICCR technology reports GPCR conformational changes occurring between the orthosteric ligand-binding site and the G protein-binding site of the receptor through an electrical signal generated by the fused Kir6.2 channel. This tool does not require activation of intracellular pathways or labeled ligands and is complementary to existing ligand binding and intracellular signaling assays. The results of this study demonstrate the ability of the ICCR technology to assess, at the receptor level, the functional cholesterol-dependence of GPCRs in a cellular environment. This assay offers new opportunities to functionally characterize ambiguous or unknown cholesterol dependence of GPCRs.

The effect of cholesterol on OXTR has been clearly previously demonstrated (68) as a positive allosteric modulation of orthosteric ligand binding. This allosteric modulation is related to the stabilization of a high-affinity state of OXTR that has a different conformation than the low affinity state (37). In this work, we discovered the reciprocity of this allosteric mechanism. Orthosteric ligands also act as positive allosteric modulators on cholesterol binding resulting in a stable interaction of functional cholesterol molecules in ligand-bound OXTRs. Thus, the presence of ligands during cholesterol-depletion or modification could have an impact in the interpretation of results in studies of cholesterol-dependence of GPCRs.

Based on published evidence, two mechanistic models can explain the cholesterol stabilization by ligand-bound OXTR. It has been shown that OXTR exists in two affinity states corresponding to two different conformations (26). Cholesterol depletion decreases the affinity for oxytocin by almost two orders of magnitude (Kd = 131 nM vs. 1.5 nM) in cholesterol-depleted guinea pig myometrium cell membrane (67). The high- and low-affinity states of OXTR (in high and low cholesterol environment respectively) co-exist in the same membrane, and the proportion of the two populations of receptors can be reversibly modified depending on the quantity of cholesterol in the membrane (67). No intermediate states were observed suggesting only two cholesterol-dependent conformations of the receptor. Cholesterol behaves as an allosteric modulator of ligand binding on OXTR not only in the cell membrane (68) but also in its solubilized form (69). These results imply intrinsic and stable interactions of cholesterol molecules with receptors even in the absence of a lipid bilayer.

**Fig. 9:**
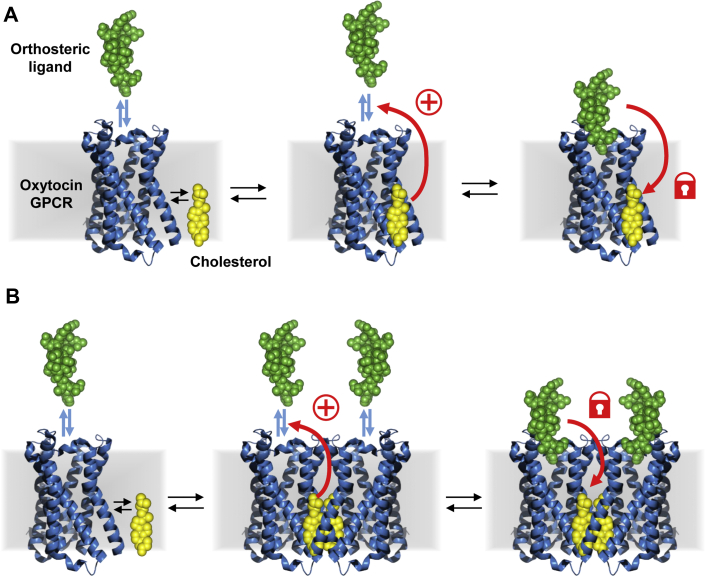
Monomeric and dimeric models of cholesterol sequestration by ligand-bound OXTR. A: Diagram showing the monomeric model of cholesterol sequestration by ligand-bound OXTR. Cholesterol and ligand binding on OXTR is in equilibrium between bound and unbound forms. Binding of cholesterol is known to induce a high-affinity state of OXTR for its ligands. In this model, binding of ligands will induce conformational changes that stabilize the binding of cholesterol and render it inaccessible to external chelators. In gray, the lipid membrane, in blue, the structure of a GPCR (M2, PDB code: 3uon) representing OXTR, in green an external orthosteric peptidic ligand (ET, PDB code: 5glh) and in yellow a cholesterol molecule (PDB code: 3d4s). B: The dimeric model showing the cholesterol-induced stabilization of OXTR homodimers which represent the high-affinity state of the receptor for its orthosteric ligands. In this model, binding of orthosteric ligands would stabilize the homodimeric form of OXTR and embed bound cholesterol molecules at the interface. GPCR, G protein-coupled receptor; OXTR, oxytocin receptor.

Molecules of cholesterol or CHS have been observed in several structures of GPCRs either in monomers or at dimer interfaces (2). Thus, the sequestered cholesterol molecules could be located either *1*) in the monomeric form of OXTR (Fig. 9A) in cavities like the one observed in the structure of the β2 adrenergic receptor (3D4S (8)) or in molecular dynamics simulations on SMO receptor (70), or *2*) at the interface of homodimers (or higher order oligomers) (71) (Fig. 9B). In the first scenario, the binding of a ligand would induce conformational changes that increase molecular interactions with bound cholesterol molecules making them less accessible to MβCD.

In the second scenario, ligand binding would stabilize a dimeric form of OXTR with cholesterol molecules at the interface. It has been shown that ligands are also able to induce oligomerization of some GPCRs such as the β2 adrenergic receptor (72). The stable, embedded position of these cholesterol molecules would prevent their accessibility to MβCD in OXTR ligand-bound state. Molecular dynamics simulations confirmed the ability of OXTR to form a known homodimeric interface (73) within the ICCR complex (supplemental Fig. S3). Both scenarios are possible, and additional approaches are required to identify the correct one. Very recently, the crystal structure of the human OXTR was obtained and published (74). In the crystallographic conditions, the structure shows a monomeric form of the receptor bound to a small molecule antagonist. Interestingly, the electron density of a cholesterol molecule is observed between the helices IV and V. This position is very close to the position observed in the model of the supplemental Fig. S3B and suggests that a cholesterol molecule at this position could be trapped in the dimeric form of the model. Additional cryo-electron microscopy structures of OXTR in lipid bilayers enriched and depleted in cholesterol and in absence and presence of ligands would also be of interest to explore in conditions closer the physiological membranes, potential new conformations as suggested by the present article.

New prospects arise for clearly identifying the binding site(s) of functional cholesterol molecules and for understanding the molecular mechanism of the dependence of OXTR on cholesterol, thanks to the possibility of selectively stabilizing the interaction of these molecules with the receptor. Thus, the simple addition of ligands during cholesterol depletion allows the removal of the bulk and nonfunctional cholesterol molecules while the interactions of the functional molecules are preserved. Associated with photoreactive cholesterol compounds (8), mass spectrometry (75), or structural approaches, this method should facilitate the identification of all binding sites of functional cholesterol. The oxytocin-induced cholesterol-bound state of OXTR appeared to be stable for more than 3 h, which is mandatory for structural studies. This finding has also potential applications in functional studies of intracellular activation and recycling of cholesterol-dependent GPCRs because the agonist-induced internalization should stabilize cholesterol interaction with the ligand-bound receptors.

In conclusion, these results reveal not only a new ICCR technology operational in living cells to characterize the cholesterol-dependence of GPCRs but also the allosteric cross-regulation occurring between the ligand-binding and the cholesterol-binding sites of OXTR. This regulation leads to the stabilization of the functional cholesterol molecules by the ligand-bound receptors.

## Data availability

The authors declare that the data supporting the findings of this study are available within the article and its supplementary information files.

## Supplemental data

This article contains supplemental data.

## Conflict of interest

The authors declare that they have no conflicts of interest with the contents of this article.
