# Supplementary material for: The ligand-bound state of a G protein-coupled receptor stabilizes the interaction of functional cholesterol molecules
Source: J Lipid Res. 2021 Feb 26;62:100059. doi: 10.1016/j.jlr.2021.100059 (PMC8050779; doi:10.1016/j.jlr.2021.100059)
Supplement: Supplemental Fig. S1 [file mmc1.pdf]

## Supplementary Figure S1: Cholesterol quantification

A)

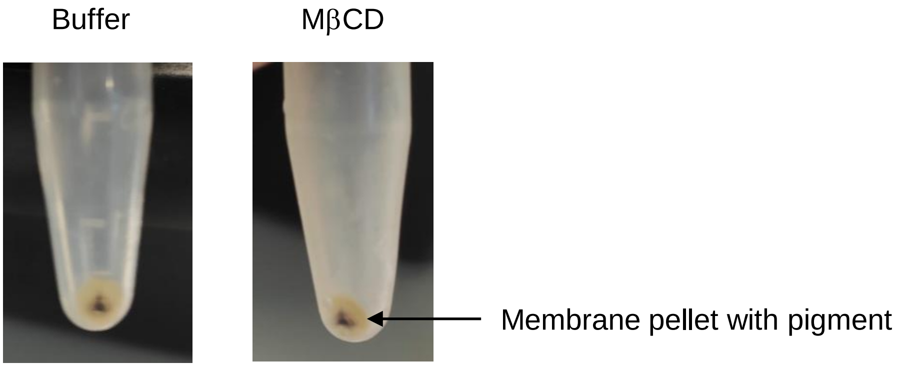


B)

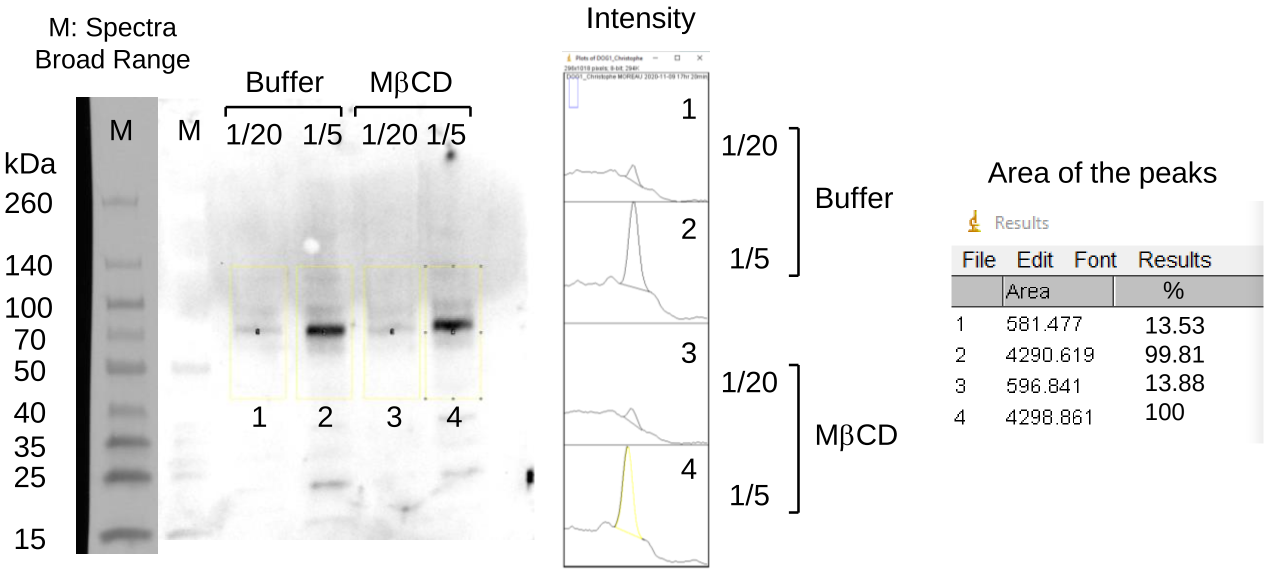


C)

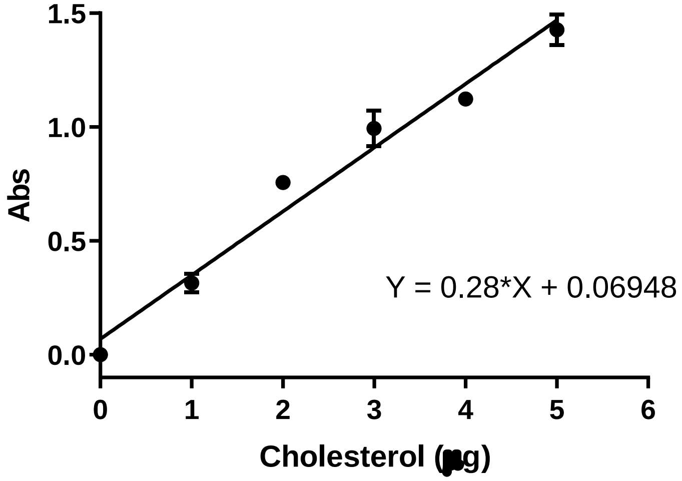


| | Absorbance | | Average Abs | Av Abs - Blank | µg |
|---|---|---|---|---|---|
| **Buffer** | 1,018 | 1,397 | 1,208 | 1,128 | **3,780** |
| **MβCD** | 0,619 | 0,953 | 0,786 | 0,707 | **2,275** |
