# Supplementary material for: The ligand-bound state of a G protein-coupled receptor stabilizes the interaction of functional cholesterol molecules
Source: J Lipid Res. 2021 Feb 26;62:100059. doi: 10.1016/j.jlr.2021.100059 (PMC8050779; doi:10.1016/j.jlr.2021.100059)

Supplemental Figure S2: Experimental conditions of TR-FRET experiments on wt OXTR expressed in mammalian cells

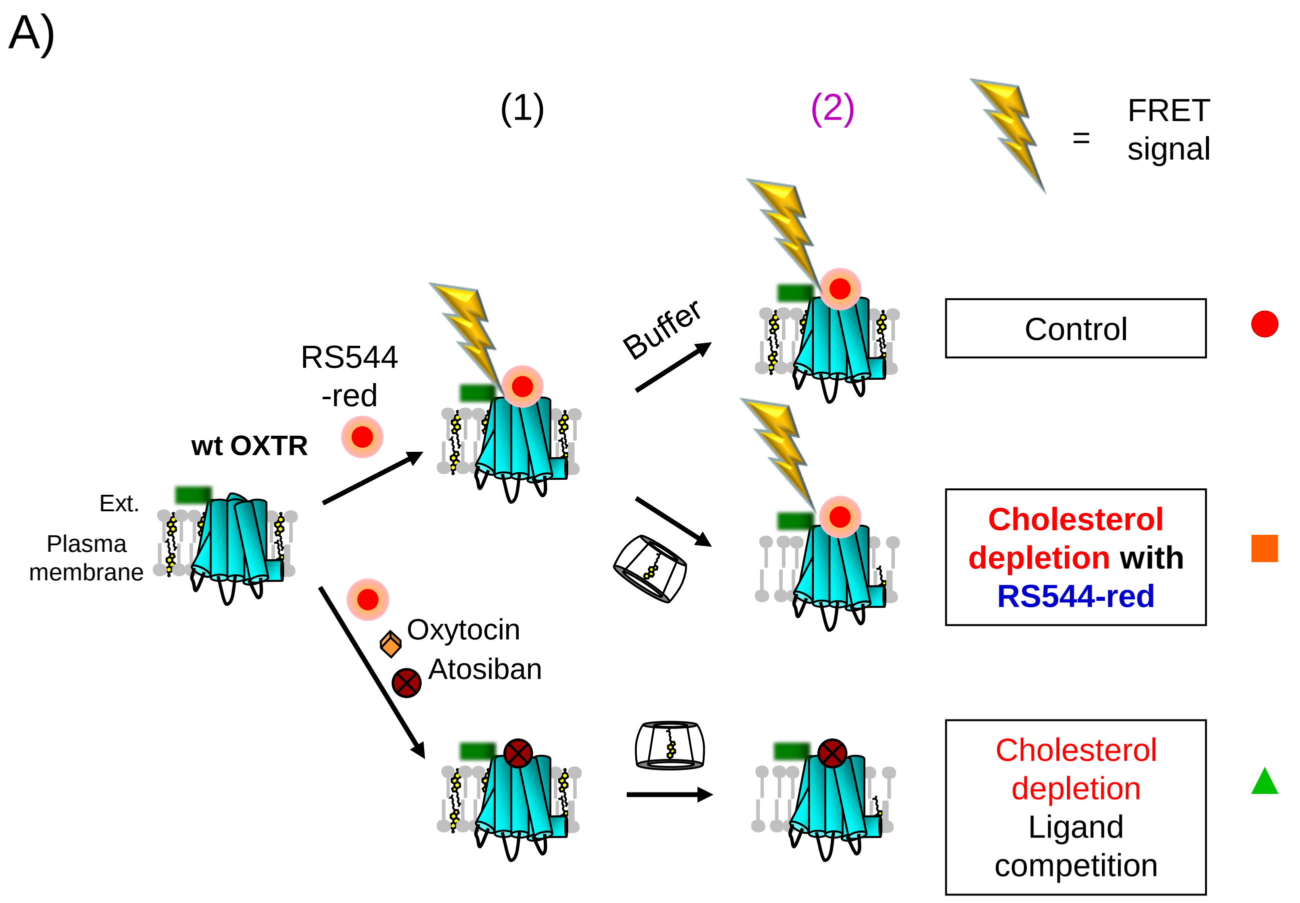

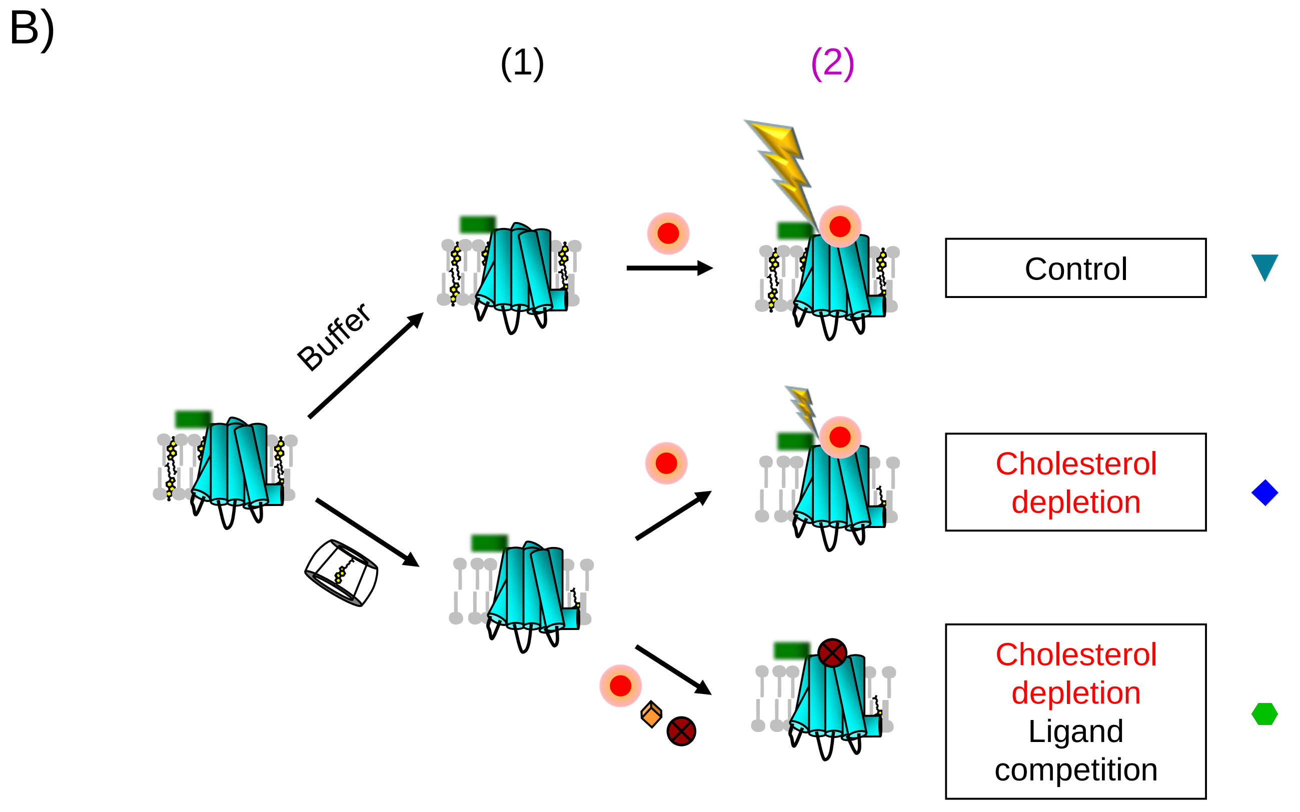

Supplement: Supplemental Fig. S2 [file mmc2.pdf]
