# Supplementary material for: The ligand-bound state of a G protein-coupled receptor stabilizes the interaction of functional cholesterol molecules
Source: J Lipid Res. 2021 Feb 26;62:100059. doi: 10.1016/j.jlr.2021.100059 (PMC8050779; doi:10.1016/j.jlr.2021.100059)

Supplementary Figure S3: Model of a OXTR dimer within the ICCR complex

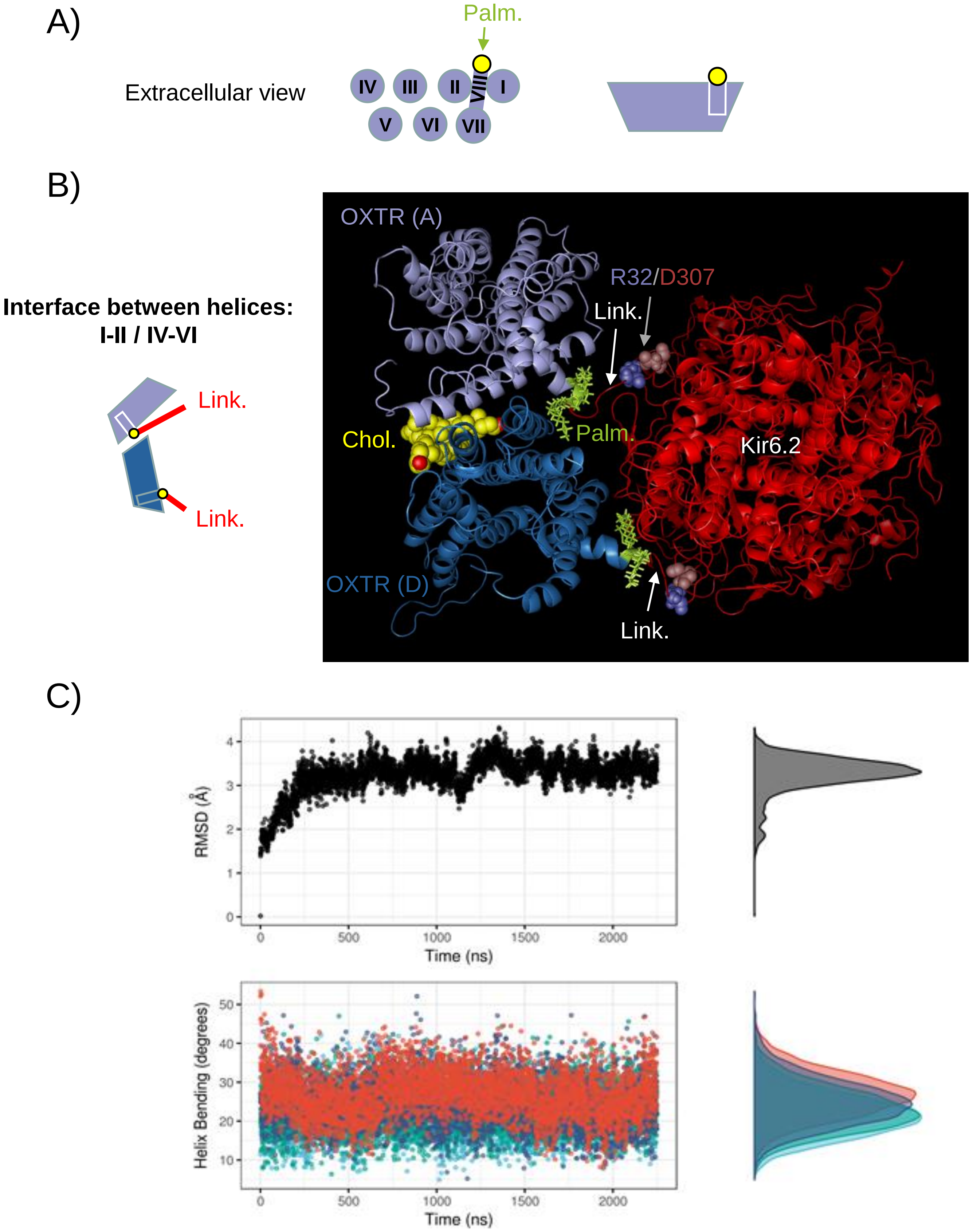

Supplement: Supplemental Fig. S3 [file mmc3.pdf]
