# Supplementary material for: The ligand-bound state of a G protein-coupled receptor stabilizes the interaction of functional cholesterol molecules
Source: J Lipid Res. 2021 Feb 26;62:100059. doi: 10.1016/j.jlr.2021.100059 (PMC8050779; doi:10.1016/j.jlr.2021.100059)
Supplement: Supplemental data [file mmc4.docx]

**SUPPLEMENTAL INFORMATION:**

**The ligand-bound state of a G protein-coupled receptor stabilizes the interaction of functional cholesterol molecules**

Laura Lemel ^a,1^, Katarzyna Nieścierowicz ^a,1,2^, M. Dolores García-Fernández ^a^, Leonardo Darré ^b^*,* Thierry Durroux ^c^, Marta Busnelli ^d^, Mylène Pezet ^e^, Fabrice Rebeille ^f^, Juliette Jouhet ^f^, Bernard Mouillac ^c^, Carmen Domene ^g^, Bice Chini ^d^, Vadim Cherezov ^h^, Christophe J Moreau ^a,^*

^a^ Univ. Grenoble Alpes, CNRS, CEA, IBS, F-38044 Grenoble, France

^b^ Functional Genomics Laboratory and Biomolecular Simulations Laboratory, Institut Pasteur de Montevideo, Montevideo, Uruguay

^c^ Institut de Génomique Fonctionnelle, CNRS, INSERM, Université de Montpellier, 141 rue de la cardonille, 34094, Montpellier Cedex 05, France

^d^ CNR, Institute of Neuroscience, Via Follerau 32, Vedano al Lambro, (MB), Italy

^e^ Institute for Advanced Biosciences, Inserm U 1209, CNRS UMR 5309, Grenoble Alpes University, Grenoble, France

^f^ Laboratoire de Physiologie Cellulaire Végétale, Université Grenoble Alpes, CNRS, CEA, INRA; F-38054, Grenoble Cedex 9, France

^g^ Department of Chemistry, University of Bath, 1 South Building, Claverton Down, Bath BA2 7AY, UK

Chemistry Research Laboratory, Mansfield Road, University of Oxford, Oxford OX1 3TA, UK

^hi^ Bridge Institute, Department of Chemistry, University of Southern California, Los Angeles, California 90089, USA.

|  |
| --- |
| **Supplemental Fig. S1. Cholesterol quantification**.  A: Pellets of plasma membranes from 600 oocytes incubated with Buffer or with 20 mM MβCD overnight. The pellets were obtained after centrifugation of homogenates at 1000 g for 15 min. and the supernatant twice at 150,000 g for 1 h at 4°C. B: Western-blot of plasma membrane samples using antibodies directed against Xenopus calcium-activated chloride channels and revealed by colorimetric digital detection using HRP substrate. Samples at two different dilutions migrated on a gradient SDS-PAGE (4-20%) gel. Band intensities were quantified with ImageJ software and the area of each peak is calculated. C: Enzymatic cholesterol quantification using a commercial kit and a standard curve. Quantity of cholesterol in Buffer and MβCD samples is estimated from the absorbance of duplicates and the equation of the standard curve. |

|  |
| --- |
| **Supplemental Fig. S2.**  Experimental conditions of TR-FRET experiments on wt OXTR expressed in mammalian cells.  A: Diagram representing the conditions of Time-Resolved FRET (TR-FRET) experiments related to the results shown in Figure 5. CHO cells expressing wtOXTR with SNAP tag in N-terminus are incubated in Step 1 either with 20 nM RS544-red, generating a FRET signal, or with 20 nM RS544-red + 5 µM unlabeled Oxytocin + 5 µM unlabeled Atosiban for determining the amplitude of the non-specific FRET signal. In Step 2, modified Barth’s solution (Buffer) or 40 mM MβCD are added. The symbols are those used in Figure 8 and the experimental conditions. B: In another set of experiments, the same solutions are used in a reverse order of Steps 1 and 2. |

|  |
| --- |
| **Supplemental Fig. S3.**  Model of an OXTR dimer within the ICCR complex.  A: Extracellular view of simplified scheme of helix organization of GPCRs. B: Extracellular view of the model of OXTR dimer with an interface between helices I-II/IV-VI reported as a potential cholesterol-related interface in GPCRs (1). Only 2 on 4 of OXTR (in blue) are shown for clarity. In the model, the subunit A (OXTR (A)) forms a dimer with the subunit D (OXTR (D)). The pore formed by the homotetramer of Kir6.2 is shown in red. The interaction of R32/D307 (Kir6.2) (2) used as constrain is shown in sphere. The last 42 residues from OXTR and the first 25 residues from Kir6.2 are truncated. The linker (Link.) is indicated and corresponds to the Kir6.2 N-terminal sequence from residue 26 to 32 (Kir6.2). Palmitoyl moieties (Palm) are shown in lemon sticks and indicate the position of the tandem of Cysteines at the end of helix VIII. Cholesterol (Chol. in yellow) molecules were observed at the dimer interface. C: Dimer structural stability. Top: backbone RMSD of the fused OXTR dimer (excluding the highly flexible linker between helix V and helix VI) from 2.25 μs of coarse grain MD simulation. Bottom: degrees of bending of helix-VIII measured using the helical maximum bending (from Bendix tool (3)) for the dimerized subunits (A: cyan and D: red) and the free subunits (B: green, C: blue). Helix-VIII bending in the dimeric subunits sample has similar values as those of the free subunits. |

# **Supplemental Material and Methods:**

Plasma membrane isolation and lipid extraction

Six hundred oocytes were incubated overnight with 20 mM MβCD or with Buffer at 19°C on an orbital shaker at 30 rpm. The protocol of plasma membrane extraction was adapted from (4). Oocytes were broken with Tris-HCl 20 mM pH 8 (10 μl/oocyte) by pipetting and the homogenates were centrifuged at 1000 g for 15 min. The supernatant was harvested and centrifuged at 150,000 g for 1 h at 4°C. The pellet obtained from the 600 oocytes per condition was resuspended in 6 ml of Tris-HCl 20 mM pH 8 supplemented with NaCl 1 M and centrifuged at 150,000 g for 1 h at 4°C. The pellet obtained was resuspended in 600 µl of Tris-HCl 20 mM pH 8. From the 600 μl of total volume, 300 μl were stored at -80°C to perform the plasma membrane protein quantification using Western-blot. Two hundred microliters of Tris-HCl 20 mM were added to the remaining 300 µl of samples. Eight hundred fifty microliters of chloroform : methanol (1:2, v:v) were added to the 500 µl of samples and vortexed 30 s. Three hundred microliters of chloroform were added again. The samples were incubated 10 min at room temperature and centrifuged 10 min at 1000 g at room temperature to separate the organic and aqueous phases. The organic phase (lower phase) containing the lipids was collected using a glass Pasteur pipette and transferred in a clean glass tube. A second extraction was performed by adding 300 μl of chloroform to the remaining aqueous phase followed by the same protocol of phase separation. Both organic phases from the same sample (MβCD or Buffer) were pooled. The organic solvents were dried by blowing argon on top of the liquid.

Western-blot on extracted plasma membranes

From the 300 µl frozen samples incubated either with MβCD or Buffer, two dilutions were prepared: 1) 1/5 : 9.6 µl of sample + 30.4 µl of Tris HCl 20 mM pH 8 + 8 µl of reducing Laemmli loading buffer (6x); and 2) 1/20: 2.4 µl of sample + 37.6 µl of Tris HCl 20 mM pH 8 + 8 µl of reducing Laemmli loading buffer (6x). No heating was performed to avoid membrane protein aggregation. Samples were loaded on Biorad pre-cast gradient SDS-polyacrylamide gel (4-20%) and migrated 1h under 200 V in standard migration buffer. Transfer to nitrocellulose membranes was performed with the Biorad Trans-Blot Turbo Transfer System. Immunostaining followed standard protocol with PBS-tween (0.1% v/v), skimmed milk 5% for saturation and 0.5% for antibody binding and washings. Primary rabbit antibody (DOG-1 polyclonal antibody, Invitrogen # PA5-87947) directed against the endogenous calcium-activated chloride channel and present in the plasma membrane was diluted 1/3000. The second goat anti-rabbit antibody conjugated to the horseradish peroxidase (Merck # 12-348) was diluted 1/5000. Both antibodies were incubated 1h at room temperature. Chemiluminescence detection was performed with the Biorad Clarity Western ECL substrates and digital imaging with the Biorad Chemidoc XRS+ imaging system. The exposure time was selected to 23.2 sec to obtain the best contrast while avoiding signal saturation.

Cholesterol quantification

Cholesterol quantification was performed with the Cholesterol Quantitation Kit (Sigma-Aldrich, MK043) according to the supplier's specifications for colorimetric detection of free acid cholesterol. Briefly, dried lipids were dissolved with 200 µl of Cholesterol Assay Buffer and vortexed, Duplicates were created by adding 50 µl of the dissolved lipid sample in two wells. Cholesterol Standards are performed according to the instructions using the Cholesterol Standard Solution. Fifty microliters of Reaction Mix is added per well and the plate is incubated in the dark for 1h at 37°C. Absorbance is measured at 570 nm with a plate-reader. Cholesterol quantity in samples is determined from the standard curve.

Method of molecular modelling

The initial configuration of the oxytocin ICCR 3D model was built combining the structure of the Kir6.2 channel published by Principalli et al. (5) and the structure of the cholesterol dependent oxytocin receptor published by Busnelli et al. (6) To obtain an ICCR structure consistent with the experimental construct, the 42 C-terminal residues of OXTR were deleted, ending with the tandem of cysteine residues in C-ter of helix VIII, and the 25 N-terminal residues of the Kir6.2 were deleted, thus generating a linker segment with sequence ...RFLCC_317_Y_26_RTRE… (shifting the numbering of the fused Kir6.2 by +292). The modified OXTR structure was manually placed next to one of the channel subunits so that OXTR C317 and Kir6.2 Y26 (Y318 in the fused protein) are at bond distance. This was repeated for each of the four subunits in the ICCR. Four PIP_2_ molecules were kept in the ICCR according to their position in the original Kir6.2 channel structure (5). Finally, the system was embedded in a lipid bilayer (the channel axis aligned to the bilayer normal, and the trans-membrane domains of the channel and OXTRs aligned with the bilayer) of composition POPC(80%):cholesterol(20%), solvated, and K^+^/Cl^-^ ions were added to neutralize the system and to achieve an ionic concentration of 150 mM. This setup was energy minimized in 20 000 steps of geometry optimization, applying position restraint on the protein backbone except for: (i) helix VIII in the fused OXTR where distance restraints were applied to ensure an α-helix conformation, and (ii) the ICCR linker (fused Kir6.2 N-terminal domain) which is free to relax. Two additional distance restraints were applied between residues R324/D599 and R326/E600 (corresponding to R32/D307 and R34/E308 in Kir6.2, respectively) to ensure experimentally observed salt bridges (5). Position restraints were also applied to PIP_2_ molecules. The CHARMM36m (7) force field was used to represent the protein, CHARMM36 (8) was used for the lipids, the TIP3P model (9) for water, and the standard CHARMM and NBFIX parameters were used for ions (10)^,^ (11). Parameters for PIP_2_ molecules were as proposed in Lupyan et al. (12). The particle mesh Ewald algorithm (13) (grid spacing of 1Å) was used for long-range electrostatic interactions and van der Waals forces were smoothly switched off between 10-12 Å. Geometry optimization calculations were performed using the NAMD2.10 software (14).

The relaxed atomistic model of the ICCR was transformed to a coarse-grained (CG) representation using the martinize.py script (version 2.6 (15)), and the MARTINI2.2 force field (15)^,^ (16) supplemented with the EnNeDyn elastic network model (17). The CG ICCR was embedded in a CG POPC(80%):cholesterol(20%) lipid bilayer (18)^,^ (19)^,^ (20)^,^ (21) and solvated with a CG aqueous electrolytic solution of ionic concentration ~150 mM. CG PIP_2_ (22) molecules were kept in the corresponding positions from the relaxed atomistic system. All CG calculations were performed using GROMACS 5.1.5 (23)^,^ (24) and the corresponding standard MARTINI simulation options. The CG system was energy minimized (5000 steps) and equilibrated (5 ns) applying position restraints on the protein backbone beads. Subsequently, a 250-ns trajectory in the NpT ensemble was recorded at 310 K and 1 atm. In all coarse-grain simulations presented in this study the velocity-rescale thermostat (25), semi-isotropic coupling with the Parrinello-Rahman barostat (26), an integration time step of 10 fs; the reaction field coulomb type with a cut-off of 1.1 nm and relative dielectric constant of 15, and a cut-off (1.1 nm) van der Waals interactions with a potential-shift-verlet modifier were used. The salt bridges restraints mentioned above were also maintained in all coarse-grain MD simulations to ensure experimentally observed salt bridges.

To explore the dimerization of the fused OXTR subunits, constant velocity pulling simulations were carried out inducing the dimerization of two adjacent subunits (A and D) while the other two (B and C) remained free to move. Pulling simulations were initialized from the final structure of the previous CG MD simulation, and forces were applied to induce the formation of the three main reported (1) dimerization interfaces. Although each dimerization interface required specific tuning of the pulling protocol, in all cases harmonic type pulling was applied in the direction defined by the vector between the centres of mass of selected helices in the fused OXTR, using a constant rate of 0.001 nm ps^-1^, and a pulling force constant of 200 kJ mol^-1^ nm^-2^. After generating these initial dimeric structures, POPC molecules in the interface were pulled away to facilitate protein-protein contacts and MD simulations with restraints (force constant of 418 kJ mol^-1^ nm^-2^) between the respective helices in each interface were run for 20 ns. Finally, 0.5 μs production MD simulations were run without any restraints between the dimerization interfaces to test the stability of each dimeric form. Once the RMSD of the fused OXTR dimer reached a plateau, a representative frame was extracted for each interface type for back-mapping to the atomistic representation using the CHARMM-GUI tool (27)^,^ (28)^,^ (29). These back-mapped systems were modified by substituting residues C316 and C317 by their palmitoylated forms, and submitted to three stages of energy minimization using the same options as those used in the initial atomistic systems setup, but with the following restraints: (1) protein backbone during 5000 steps, (2) protein backbone except for the ICCR linker and helix VIII, combined with distance restraints on the latter to maintain the α-helix conformation, and distance restraints between residues R324/D599 and R326/E600, to ensure experimentally observed salt brides during 5000 steps, (3) only distance restraints in helix VIII and experimental salt brides during 3000 steps. To gain further statistics on the stability of the three studied dimerization interfaces, CG MD simulations were restarted after the initial 0.5 μs and extended up to 2.25 μs. Deformation of helix-VIII was measured on these extended simulations using the helical maximum bending metric calculated using the Bendix tool (3). One on the three models with the interface between helices I-II/IV-VI demonstrated no deformation of the OXTR protomer structure and is presented in the Supplemental Figure S2.

References

1. Sengupta, D., G. Kumar, and A. Chattopadhyay. 2017. Interaction of Membrane Cholesterol with GPCRs: Implications in Receptor Oligomerization | SpringerLink Humana Press, Cham.

2. Principalli, M. A., L. Lemel, A. Rongier, A. C. Godet, K. Langer, J. Revilloud, L. Darre, C. Domene, M. Vivaudou, and C. J. Moreau. 2017. Functional mapping of the N-terminal arginine cluster and C-terminal acidic residues of Kir6.2 channel fused to a G protein-coupled receptor. *Biochim Biophys Acta Biomembr* **1859**: 2144-2153.

3. Dahl, A. C., M. Chavent, and M. S. Sansom. 2012. Bendix: intuitive helix geometry analysis and abstraction. *Bioinformatics* **28**: 2193-2194.

4. Bergeron, M. J., R. Boggavarapu, M. Meury, Z. Ucurum, L. Caron, P. Isenring, M. A. Hediger, and D. Fotiadis. 2011. Frog oocytes to unveil the structure and supramolecular organization of human transport proteins. *Plos One* **6**: e21901.

5. Principalli, M. A., L. Lemel, A. Rongier, A. C. Godet, K. Langer, J. Revilloud, L. Darre, C. Domene, M. Vivaudou, and C. J. Moreau. 2017. Functional mapping of the N-terminal arginine cluster and C-terminal acidic residues of Kir6.2 hannel fused to a G protein-coupled receptor. *Bba-Biomembranes* **1859**: 2144-2153.

6. Busnelli, M., G. Kleinau, M. Muttenthaler, S. Stoev, M. Manning, L. Bibic, L. A. Howell, P. J. McCormick, S. Di Lascio, D. Braida, M. Sala, G. E. Rovati, T. Bellini, and B. Chini. 2016. Design and Characterization of Superpotent Bivalent Ligands Targeting Oxytocin Receptor Dimers via a Channel-Like Structure. *J Med Chem* **59**: 7152-7166.

7. Huang, W., A. Manglik, A. J. Venkatakrishnan, T. Laeremans, E. N. Feinberg, A. L. Sanborn, H. E. Kato, K. E. Livingston, T. S. Thorsen, R. C. Kling, S. Granier, P. Gmeiner, S. M. Husbands, J. R. Traynor, W. I. Weis, J. Steyaert, R. O. Dror, and B. K. Kobilka. 2015. Structural insights into micro-opioid receptor activation. *Nature* **524**: 315-321.

8. Klauda, J. B., R. M. Venable, J. A. Freites, J. W. O'Connor, D. J. Tobias, C. Mondragon-Ramirez, I. Vorobyov, A. D. MacKerell, Jr., and R. W. Pastor. 2010. Update of the CHARMM all-atom additive force field for lipids: validation on six lipid types. *J Phys Chem B* **114**: 7830-7843.

9. Jorgensen, W. L., J. Chandrasekhar, J. D. Madura, R. W. Impey, and M. L. Klein. 1983. Comparison of Simple Potential Functions for Simulating Liquid Water. *J Chem Phys* **79**: 926-935.

10. Beglov, D., and B. Roux. 1994. Finite Representation of an Infinite Bulk System - Solvent Boundary Potential for Computer-Simulations. *J Chem Phys* **100**: 9050-9063.

11. Luo, Y., and B. Roux. 2010. Simulation of Osmotic Pressure in Concentrated Aqueous Salt Solutions. *Journal of Physical Chemistry Letters* **1**: 183-189.

12. Lupyan, D., M. Mezei, D. E. Logothetis, and R. Osman. 2010. A molecular dynamics investigation of lipid bilayer perturbation by PIP2. *Biophys J* **98**: 240-247.

13. Essmann, U., L. Perera, M. L. Berkowitz, T. Darden, H. Lee, and L. G. Pedersen. 1995. A Smooth Particle Mesh Ewald Method. *J Chem Phys* **103**: 8577-8593.

14. Phillips, J. C., R. Braun, W. Wang, J. Gumbart, E. Tajkhorshid, E. Villa, C. Chipot, R. D. Skeel, L. Kale, and K. Schulten. 2005. Scalable molecular dynamics with NAMD. *J Comput Chem* **26**: 1781-1802.

15. de Jong, D. H., G. Singh, W. F. Bennett, C. Arnarez, T. A. Wassenaar, L. V. Schafer, X. Periole, D. P. Tieleman, and S. J. Marrink. 2013. Improved Parameters for the Martini Coarse-Grained Protein Force Field. *J Chem Theory Comput* **9**: 687-697.

16. Monticelli, L., S. K. Kandasamy, X. Periole, R. G. Larson, D. P. Tieleman, and S. J. Marrink. 2008. The MARTINI Coarse-Grained Force Field: Extension to Proteins. *J Chem Theory Comput* **4**: 819-834.

17. Periole, X., M. Cavalli, S. J. Marrink, and M. A. Ceruso. 2009. Combining an Elastic Network With a Coarse-Grained Molecular Force Field: Structure, Dynamics, and Intermolecular Recognition. *J Chem Theory Comput* **5**: 2531-2543.

18. Marrink, S. J., A. H. de Vries, and A. E. Mark. 2004. Coarse grained model for semiquantitative lipid simulations. *J Phys Chem B* **108**: 750-760.

19. Melo, M. N., H. I. Ingolfsson, and S. J. Marrink. 2015. Parameters for Martini sterols and hopanoids based on a virtual-site description. *J Chem Phys* **143**: 243152.

20. Marrink, S. J., H. J. Risselada, S. Yefimov, D. P. Tieleman, and A. H. de Vries. 2007. The MARTINI force field: coarse grained model for biomolecular simulations. *J Phys Chem B* **111**: 7812-7824.

21. Ingolfsson, H. I., M. N. Melo, T. A. Wassenaar, X. Periole, A. H. de Vries, D. P. Tieleman, and S. J. Marrink. 2015. Computational Lipidomics and the Lipid Organization of Cell Envelopes. *Biophys. J.* **108**: 342a-342a.

22. Lopez, C. A., Z. Sovova, F. J. van Eerden, A. H. de Vries, and S. J. Marrink. 2013. Martini Force Field Parameters for Glycolipids. *J Chem Theory Comput* **9**: 1694-1708.

23. Berendsen, H. J. C., D. Vanderspoel, and R. Vandrunen. 1995. Gromacs - a Message-Passing Parallel Molecular-Dynamics Implementation. *Comput Phys Commun* **91**: 43-56.

24. Van Der Spoel, D., E. Lindahl, B. Hess, G. Groenhof, A. E. Mark, and H. J. Berendsen. 2005. GROMACS: fast, flexible, and free. *J Comput Chem* **26**: 1701-1718.

25. Bussi, G., D. Donadio, and M. Parrinello. 2007. Canonical sampling through velocity rescaling. *J Chem Phys* **126**: 014101.

26. Parrinello, M., and A. Rahman. 1981. Polymorphic Transitions in Single-Crystals - a New Molecular-Dynamics Method. *J Appl Phys* **52**: 7182-7190.

27. Jo, S., T. Kim, V. G. Iyer, and W. Im. 2008. CHARMM-GUI: a web-based graphical user interface for CHARMM. *J Comput Chem* **29**: 1859-1865.

28. Qi, Y., H. I. Ingolfsson, X. Cheng, J. Lee, S. J. Marrink, and W. Im. 2015. CHARMM-GUI Martini Maker for Coarse-Grained Simulations with the Martini Force Field. *J Chem Theory Comput* **11**: 4486-4494.

29. Lee, J., X. Cheng, J. M. Swails, M. S. Yeom, P. K. Eastman, J. A. Lemkul, S. Wei, J. Buckner, J. C. Jeong, Y. Qi, S. Jo, V. S. Pande, D. A. Case, C. L. Brooks, 3rd, A. D. MacKerell, Jr., J. B. Klauda, and W. Im. 2016. CHARMM-GUI Input Generator for NAMD, GROMACS, AMBER, OpenMM, and CHARMM/OpenMM Simulations Using the CHARMM36 Additive Force Field. *J Chem Theory Comput* **12**: 405-413.
